# Inhibiting amyloid beta (1–42) peptide-induced mitochondrial dysfunction prevents the degradation of synaptic proteins in the entorhinal cortex

**DOI:** 10.3389/fnagi.2022.960314

**Published:** 2022-10-06

**Authors:** Olayemi Joseph Olajide, Claudia La Rue, Andreas Bergdahl, Clifton Andrew Chapman

**Affiliations:** ^1^Department of Psychology, Center for Studies in Behavioral Neurobiology, Concordia University, Montreal, QC, Canada; ^2^Division of Neurobiology, Department of Anatomy, College of Health Sciences, University of Ilorin, Ilorin, Nigeria; ^3^Department of Health, Kinesiology and Applied Physiology, Concordia University, Montreal, QC, Canada

**Keywords:** Alzheimer’s disease, mitochondria, acetylcholine, entorhinal cortex, oxidative stress, reactive oxygen species, synaptic proteins

## Abstract

Increasing evidence suggests that mitochondrial dysfunction and aberrant release of mitochondrial reactive oxygen species (ROS) play crucial roles in early synaptic perturbations and neuropathology that drive memory deficits in Alzheimer’s disease (AD). We recently showed that solubilized human amyloid beta peptide 1–42 (hAβ_1–42_) causes rapid alterations at glutamatergic synapses in the entorhinal cortex (EC) through the activation of both GluN2A- and GluN2B-containing NMDA receptors. However, whether disruption of mitochondrial dynamics and increased ROS contributes to mechanisms mediating hAβ_1–42_-induced synaptic perturbations in the EC is unknown. Here we assessed the impact of hAβ_1–42_ on mitochondrial respiratory functions, and the expression of key mitochondrial and synaptic proteins in the EC. Measurements of mitochondrial respiratory function in wild-type EC slices exposed to 1 μM hAβ_1–42_ revealed marked reductions in tissue oxygen consumption and energy production efficiency relative to control. hAβ_1–42_ also markedly reduced the immunoexpression of both mitochondrial superoxide dismutase (SOD2) and mitochondrial-cytochrome c protein but had no significant impact on cytosolic-cytochrome c expression, voltage-dependent anion channel protein (a marker for mitochondrial density/integrity), and the immunoexpression of protein markers for all five mitochondrial complexes. The rapid impairments in mitochondrial functions induced by hAβ_1–42_ were accompanied by reductions in the presynaptic marker synaptophysin, postsynaptic density protein (PSD95), and the vesicular acetylcholine transporter, with no significant changes in the degradative enzyme acetylcholinesterase. We then assessed whether reducing hAβ_1–42_-induced increases in ROS could prevent dysregulation of entorhinal synaptic proteins, and found that synaptic impairments induced by hAβ_1–42_ were prevented by the mitochondria-targeted antioxidant drug mitoquinone mesylate, and by the SOD and catalase mimetic EUK134. These findings indicate that hAβ_1–2_ can rapidly disrupt mitochondrial functions and increase ROS in the entorhinal, and that this may contribute to synaptic dysfunctions that may promote early AD-related neuropathology.

## Introduction

Alzheimer’s disease (AD) is a debilitating neurodegenerative disorder that results from the progressive loss of neurons in selective brain areas, and is the primary cause of dementia worldwide (Fan et al., 2020; Guo et al., 2020; Olajide et al., 2021). Neuropathology in AD is hallmarked by abnormal accumulation of amyloid beta peptide (Aβ), a highly neurotoxic derivative of the amyloid precursor protein (APP) which is a transmembrane protein that is particularly concentrated in synapses (Haass and Selkoe, 2007; Masters and Selkoe, 2012; Mucke and Selkoe, 2012; Hampel et al., 2021). Aβ is thought to interact with multiple biological mechanisms to incite AD-related modifications in neurons, and several lines of experimental evidence, including our own, have shown that Aβ can rapidly dysregulate proteins mediating excitatory synaptic transmission (Selkoe, 2002; Coleman and Yao, 2003; Shankar and Walsh, 2009; Overk and Masliah, 2014; Tu et al., 2014; Olajide and Chapman, 2021; Olajide et al., 2021). These synaptic changes are thought to lead to cognitive deficits and memory failure early in AD (Arendt, 2009; Mucke and Selkoe, 2012; Marsh and Alifragis, 2018; Guo et al., 2020), but the molecular mechanisms through which Aβ induces excitotoxicity and neurodegeneration are still poorly understood.

Synaptic transmission relies heavily on mitochondria that generate energy through ATP and nicotinamide adenine dinucleotide (NAD +), maintain calcium homeostasis and buffering, and regulate cell signaling (Calkins et al., 2011; Akhter et al., 2017; Pickett et al., 2018). Perturbation of mitochondrial functions may contribute directly to impaired synaptic transmission in early AD (Cavallucci et al., 2013; Akhter et al., 2017; Olajide et al., 2017a; Xiao et al., 2017; Pickett et al., 2018; Khosravi and Harner, 2020), and Aβ can disrupt mitochondrial energy production and lead to increases in the synthesis of reactive oxygen species (ROS) and oxidative damage (Calkins et al., 2011; Hampel et al., 2021; Olajide et al., 2021; Ashleigh et al., 2022). Cellular ROS are natural by-products of mitochondrial aerobic respiration that result predominantly from leakage of electrons at complexes I and III of the electron transport chain. While ROS serve essential cellular functions, excess ROS can induce oxidative damage and AD-related neuropathology (Murphy, 2009; Patten et al., 2010; Wang et al., 2020). Under physiological conditions, oxidative damage by the major ROS, superoxide (O^2^−), is prevented by mitochondrial superoxide dismutase (SOD2), an antioxidant enzyme that catalyzes the dismutation of O^2^− to hydrogen peroxide (Murphy, 2009). However, Aβ can induce mitochondrial dysfunction both by increasing O^2^− production and by depleting the cellular antioxidant defense system, which causes degeneration of synaptic elements and alters neurotransmission processes in neurons (Tönnies and Trushina, 2017; Wang et al., 2020; Ionescu-Tucker and Cotman, 2021; Misrani et al., 2021). The overexpression of SOD2 in AD mouse models reportedly reduces Aβ deposition and prevents memory deficits (Dumont et al., 2009; Massaad et al., 2009), whereas mutant AD mice with depleted SOD2 expression show increased Aβ levels and accelerated synaptic dysfunction and cognitive decline (Li et al., 2004; Esposito et al., 2006).

The entorhinal cortex (EC) is important for cognitive functions including memory and is among the first cortical regions to be affected by AD pathology (van Hoesen et al., 1991; Velayudhan et al., 2013; Khan et al., 2014; Zhou et al., 2016; Grubman et al., 2019; Olajide et al., 2021). Subpopulations of neurons are differentially vulnerable to the toxicity of oxidative stress (Wang and Michaelis, 2010), and the EC is one of the earliest temporal lobe structures to show both oxidative impairment and Aβ accumulation (Nunomura et al., 2001; Terni et al., 2010; Olajide et al., 2021). Armand-Ugon et al. (2017) reported reduced expression of mitochondrial complexes I, II, IV, and V in the EC, but not in the frontal cortex, during the initial stages of AD, and this mitochondrial dysfunction and oxidative impairment may drive early synaptic failure in AD (Tönnies and Trushina, 2017). We have shown recently that human Aβ_1–42_ (hAβ_1–42_) rapidly alters elements of glutamatergic synapses in the EC through activation of both GluN2A and GluN2B subunit-containing NMDA receptors (Olajide and Chapman, 2021). This may drive the selective vulnerability of the EC to AD-type neurodegeneration (Olajide et al., 2021), but whether increased ROS and mitochondrial dysfunction contribute to the susceptibility of EC to early AD-related synaptic degeneration has not been explored.

Here, we investigated how mitochondrial proteins, respiratory function, and key synaptic proteins are affected in the EC of brain slices from wild-type rats following exposure to soluble hAβ_1–42_. We then assessed the ability of two novel and specific pharmacological ROS scavengers and inhibitors, the mitochondria-targeted antioxidant mitoquinone mesylate (MitoQ) and the SOD and catalase mimetic EUK134, to block the degradation of synaptic proteins induced by hAβ_1–42_ in the EC. Our results support the idea that mitochondrial alterations induced by hAβ_1–42_ are a central factor in early synaptic degeneration in the EC, and contribute to a more detailed understanding of the molecular mechanisms driving neurodegeneration in the EC during early AD.

## Materials and methods

### Animals and tissue preparation

Experiments were conducted according to the guidelines of the Canadian Council on Animal Care, and experimental procedures were approved by the Concordia University Animal Research Ethics Committee (Permit Number: 30000253). Six to ten-week-old male Long-Evans rats (Charles River) were housed and placed on a reverse 12 h: 12 h light-dark cycle, with free access to rat chow and water. Acute brain slices were obtained following isoflurane anesthesia and decapitation as previously described (Glovaci and Chapman, 2019). Brains were rapidly removed and cooled (4°C) in high-sucrose ACSF containing (in mM) 250 sucrose, 2 KCl, 1.25 NaH_2_PO_4_, 7 MgCl_2_, 26 NaHCO_3_, 0.5 CaCl_2_ and 10 dextrose, saturated with 95% O_2_ and 5% CO_2_. Horizontal slices (400 μm thick) were obtained throughout the ventral to the dorsal extent of the brain in cooled high-sucrose ACSF using a vibratome (Leica, VT1200). The EC was carefully excised from each slice, using a flat blade in contact with the vibratome blade (Paxinos and Watson, 1997; Olajide and Chapman, 2021). Obtained tissue was placed in normal ACSF consisting (in mM) of 124 NaCl, 5 KCl, 1.25 NaH_2_PO_4_, 2 MgSO_4_, 2 CaCl_2_, 26 NaHCO_3_, and 10 dextrose saturated with 95% O_2_ and 5% CO_2_ at 32°C for 30 min. Assignment of EC slices taken from the right or left hemispheres were alternated between treatment groups at each consecutive level, so that tissue in the control and treated conditions was obtained from both hemispheres across the dorso-ventral extent of the EC.

### Drug preparation and treatments

Drugs were prepared as stock solutions and diluted to final concentrations just before use. Tissue was incubated for 3 h in submersion chambers containing ACSF at 22–24°C, saturated with 95% O_2_ and 5% CO_2_. EC slices were incubated in 1 μM hAβ_1–42_ (MW 4514.08; Abcam, AB120301) with a final concentration of DMSO of 0.1%, while control EC slices were exposed to 0.1% DSMO in ACSF. Solubilization and preparation of hAβ_1–42_ were done as previously described (Olajide and Chapman, 2021). hAβ_1–42_ was first solubilized in DMSO at 500 μM, sonicated for 15 min at room temperature, and then centrifuged at 15,000 × g at 4°C for 20 min. The supernatant was stored at −80°C in 10 μL aliquots and diluted in ACSF just before use. This method is known to result in low molecular weight β-oligomers including monomers to tetramers (Bitan et al., 2003; Stine et al., 2003) which are the most neurotoxic (Masters and Selkoe, 2012; Guo et al., 2020). It is possible for some protofibrils to develop following this preparation, but significant fibrillary aggregation requires longer incubation times and higher concentrations (>10 μM; O’Nuallain et al., 2004; Wogulis et al., 2005).

The role of mitochondrial ROS and oxidative stress was assessed by applying the mitochondria-targeted antioxidant drug mitoquinone mesylate (MitoQ, 500 nM; Toronto Research Chemicals; M372215), and SOD/catalase mimetic drug EUK134 (250 nM; Cayman Chemical; 10006329) during incubation of slices in hAβ_1–42_ or DMSO. Each treatment group included slices from at least 6 animals.

### Mitochondrial respiration measurements

A sequential substrate addition protocol was conducted to assess mitochondrial coupled and uncoupled oxygen consumption, LEAK respiration, and membrane integrity using a two-chamber polarographic sensor (Oxygraph-2k; Oroboros Instruments, Innsbruck, Austria). hAβ_1–42_-incubated and control EC samples were assessed simultaneously in both chambers under similar reaction conditions (*n* = 5 or 6). The measurements of oxygen consumption were performed in MiR05 at 37^°^C. MiR05 contains (in mM) 0.5 EGTA, 3.0 MgCl_2_⋅6H_2_O, 60 K-lactobionate, 20 taurine, 10 KH_2_PO_4_, 20 HEPES, 110 sucrose, and 1 g/L BSA (pH 7.1). The oxygen flux was registered and analyzed by the DatLab 7.0 software. Between 2 and 3 mg (wet weight) of treated entorhinal tissue was placed in the Oxygraph and oxygen levels were increased to approximately 480 pmol. Saponin (50 μg/mL) was added to the chambers to permeabilize the tissue before the experiment was begun. In the protocol, non-phosphorylating LEAK-respiration was induced by adding the Complex I-linked substrates malate (2 mM), pyruvate (5 mM), and glutamate (5 mM). Subsequently, the OXPHOS-capacity of Complex I-linked activity was measured after the addition of a saturating concentration of ADP (5 mM). Cytochrome c (10 μM) was then added to assess mitochondrial membrane damage. OXPHOS-capacity with combined Complex I and II-linked substrates was assessed by the addition of succinate (10 mM). This was followed by FCCP (carbonylcyanide-4 (trifluoromethoxy) phenylhydrazone, 1 μM) to test for uncoupling. The acceptor control ratio (ACR), which measures the degree of coupling between oxidation and phosphorylation, was calculated as the rate of oxygen consumption during ADP phosphorylation divided by the rate of non-phosphorylating LEAK-respiration induced by glutamate administration.

### Protein preparation and Western blotting

To perform Western immunoblotting, treated EC tissue was collected into microfuge tubes and snap-frozen. Tissues were disrupted with a tissue sonicator (QSonica: Q55) in homogenization buffer (10 mM Tris, pH 7.4, 1 mM EGTA, 1 mM EDTA, 0.5 DTT, 0.1 μM okadaic acid, 1 mM Na_3_VO_4_, 1 mM PMSF and 10 μg/mL leupeptin). Mitochondrial protein purification was done following extraction in mitochondrial homogenization buffer (10 mM Tris-HCl, pH 6.7, 10 mM KCl, 0.15 mM MgCl_2_, 1 mM PMSF, 1 mM DTT) followed by resuspension in mitochondrial suspension buffer (10 mM Tris HCl-pH 6.7, 0.15 mM MgCl_2_, 0.25 mM sucrose, 1 mM PMSF, 1 mM DTT). The quantity of protein in each sample was determined using BCA Protein Assay (Thermo Fisher: 23,227) and an ELISA Fluorostar Analysis System Plate Reader. Bovine serum albumin (BSA) was used as the standard for protein quantification. Protein samples (20–30 μg) were resolved on Tris-glycine 8–12% SDS-PAGE gels. The resolved proteins were transferred from gels to nitrocellulose membrane (Bio-Rad: 1620112) and blocked for 1–2 h in either 5% milk or 5% BSA (as indicated below for each specific antibody) in Tris-buffered saline (TBS) containing 0.2% Tween-20 (TBST). All antibodies used were solubilized (0.5–2.0 mg/mL) by manufacturers in buffered saline or culture supernatant containing 0.02–0.1% sodium azide (pH 7.2–7.6). Primary antibodies were diluted for overnight incubation of membranes at 4°C, and included total oxidative phosphorylation (OXPHOS) rodent antibody cocktail (1:2,000, 5% milk in TBST, MitoSciences, MS604), rabbit anti-SOD2 antibody (1:4,000, 5% milk in TBST; Proteintech, 24127-1-AP), rabbit anti-Cytochrome c antibody (1:2,000, 5% milk in TBST; Abcam, AB133504), mouse anti-VDAC-1/Porin antibody (1:2,000, 5% BSA in TBST, Abcam, AB14734), mouse anti-synaptophysin (1:3,000, 5% BSA in TBST; Sigma-Aldrich, MAB5258), rabbit anti-PSD95 (1:3,000, 5% milk in TBST; Abcam, AB18258), rabbit anti-choline acetylcholinesterase (1:1,000, 5% milk in TBST; Abcam, AB183591), rabbit anti-vesicular acetylcholine transporter (1:2,000, 5% milk in TBST; Abcam, AB235201), mouse anti-vinculin (1:4,000, 5% milk in TBST; Abcam, AB130007), and mouse anti-β-Actin (1:5,000, 5% BSA in TBST; Abcam, AB8226). Membranes were then washed 3 times for 5 min each in TBST and incubated at room temperature with either peroxidase-conjugated goat anti-mouse secondary antibody (1:4,000; Millipore Sigma, AP124P) or peroxidase-conjugated goat anti-rabbit secondary antibody (1:5,000; Millipore Sigma, AP132P) for 1–2 h. Immunoreactivity was detected using ECL Western blotting substrate (Thermo Fisher Scientific, 32106) and visualized using a CDP-STAR chemiluminescence system (Amersham hyperfilm ECL). All antibody signals were normalized against loading control (β-Actin or vinculin) immunoreactivity. Western blot data were compiled from six animals, and bands were quantified by densitometric analysis using Image-J software (version 1.41).

### Data analysis

Results obtained from mitochondrial respiratory measurements were tested statistically using a two-tailed Student’s *t*-test. Data from Western blot densitometry were analyzed using either two-tailed Student’s *t*-tests or one-way analyses of variance (ANOVA) with Sidak’s multiple comparisons tests. All data analyses were done using GraphPad Prism software version 8.0.1 with significance set at *p* < 0.05. Bar graphs indicate the mean and standard deviation, normalized to the largest control value in percentage (for protein analysis), and include plots showing values obtained from individual animals.

## Results

### Effects of hAβ_1–42_ on entorhinal mitochondrial function and integrity

**FIGURE 1:**
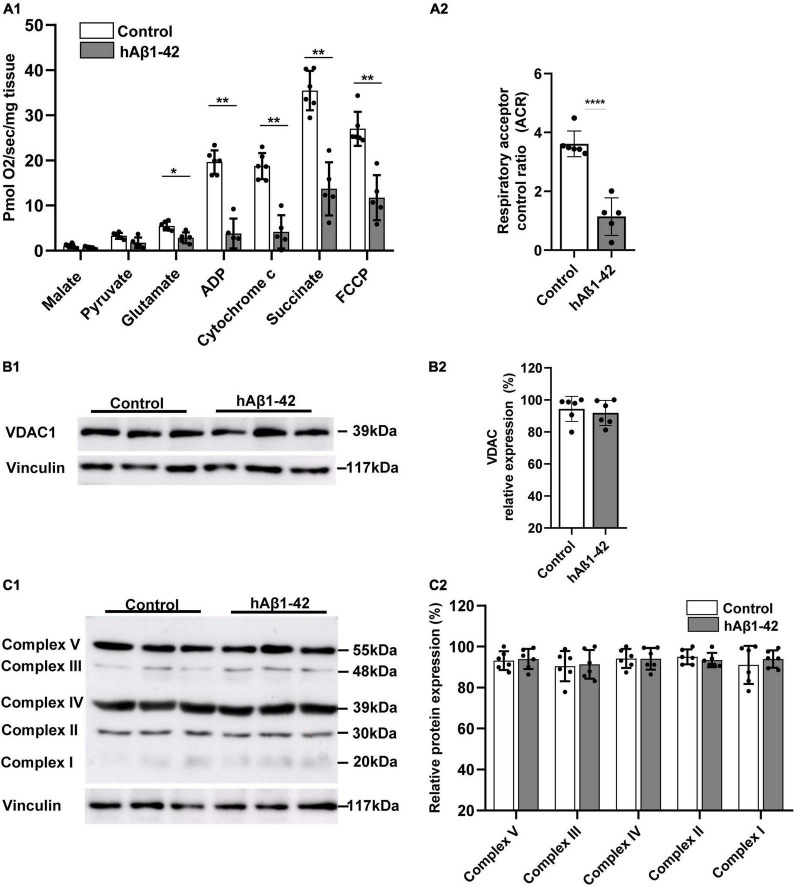
Impact of hAβ_1–42_ on mitochondrial respiration and function in the entorhinal cortex. **(A1)** Bar graphs represent mitochondrial substrate utilization in permeabilized entorhinal tissue previously treated with 1 μM hAβ_1–42_ for 3 h vs. control tissue, assessed through high-resolution respirometry (*n* = 5–6). **(A2)** Graph showing the acceptor control ratio (ACR) which measures the relative efficiency of phosphorylation and is determined by dividing ADP by glutamate average rates of respiration. hAβ_1–42_ treatment significantly reduced the ACR relative to control. **(B1)** Representative immunoblots of mitochondrial membrane and gatekeeper protein voltage-dependent anion channel 1 (VDAC1) and vinculin (loading control) in entorhinal lysates following incubation in control medium or hAβ_1–42_. **(B2)** Bar graphs showing the normalized protein expression of VDAC1 (*n* = 6). **(C1)** Representative immunoblots of mitochondrial complex I to V with total OXPHOS rodent antibody cocktail with vinculin serving as a loading control. **(C2)** Graphs showing normalized data for all five mitochondrial subunits in hAβ_1–42_-treated slices vs. the largest expression in the control group (*n* = 6) (*p < 0.05; **p < 0.01; ****p < 0.0001).

During the pathogenesis of AD, the EC selectively accumulates neurotoxic Aβ and shows the earliest signs of oxidative impairment and neurodegeneration (Terni et al., 2010; Olajide et al., 2021), but the mechanisms involved are still unclear. We therefore examined the rapid effects of hAβ_1–42_ on mitochondrial functions in entorhinal tissue using high-resolution respirometry. Utilization of respiration substrates in entorhinal samples was significantly reduced by exposing brain slices to 1 μM hAβ_1–42_ for a period of 3 h (Figure 1A1). Relative to control, hAβ_1–42_ markedly reduced respiration in EC slices (in pmol/s/mg; *n* = 5–6) following the addition of glutamate (2.9 ± 1.2 vs. 5.6 ± 1.0 in controls; *p* = 0.0211), ADP (3.8 ± 3.3 vs. 20.2 ± 2.4; *p* = 0.0022), cytochrome c (4.2 ± 3.7 vs. 19.4 ± 2.7; *p* = 0.0036), succinate (13.7 ± 5.9 vs. 36.0 ± 4.7; *p* = 0.0045), and FCCP (11.7 ± 5.0 vs. 27.0 ± 4.0; *p* = 0.0070). However, incubation of EC slices with hAβ_1–42_ did not significantly alter the consumption of malate (0.7 ± 0.2 vs. 1.1 ± 0.5 in controls; *p* = 0.2427), and pyruvate (1.8 ± 1.2 vs. 3.5 ± 0.6; *p* = 0.0570). hAβ_1–42_ also markedly reduced the ratio between oxidation and phosphorylation which measures the efficiency of the mitochondria in generating ATP per given amount of oxygen, and is shown by assessments of the ACR (1.1 ± 0.6 vs. 3.6 ± 0.4 in controls; *p* < 0.0001; Figure 1A2).

We next assessed the impact of hAβ_1–42_ on immunoexpression of markers for the integrity of the mitochondrial membrane and for the five mitochondrial protein complexes that make up the electron transport chain. Compared to control, hAβ_1–42_ treatment did not alter immunoblot expression of the mitochondrial membrane and gatekeeper protein voltage-dependent anion channel 1 (VDAC1; 91.9 ± 7.3 vs. 94.3 ± 7.8 in controls; *p* = 0.6125) (Figure 1B). Similarly, immunoblotting of complexes I to V with total OXPHOS rodent antibody cocktail (Figure 1C) revealed no significant changes (*n* = 6) in protein expression between control and hAβ_1–42_-treated entorhinal slices. When compared to control, hAβ_1–42_ (1 μM) treatment for 3 h did not alter the normalized protein expression of Complex V (93.2 ± 4.6 and 94.0 ± 5.0; *p* = 0.785), Complex III (90.5 ± 7.4 and 91.4 ± 7.0; *p* = 0.833), Complex IV (94.2 ± 4.6 and 94.1 ± 5.3; *p* = 0.961), Complex II (95.1 ± 3.6 and 93.4 ± 3.5; *p* = 0.560), and Complex I (91.1 ± 9.3 and 94.0 ± 4.3; *p* = 0.589) in entorhinal lysates. Therefore, although hAβ_1–42_ treatment reduces mitochondrial function as reflected in respirometry analysis, it did not significantly affect the expression of protein markers for mitochondrial integrity and for respiratory complexes.

### hAβ_1–42_ reduces immunoexpression of mitochondrial enzymes, synaptophysin, postsynaptic density protein-95 and vesicular acetylcholine transporter

**FIGURE 2:**
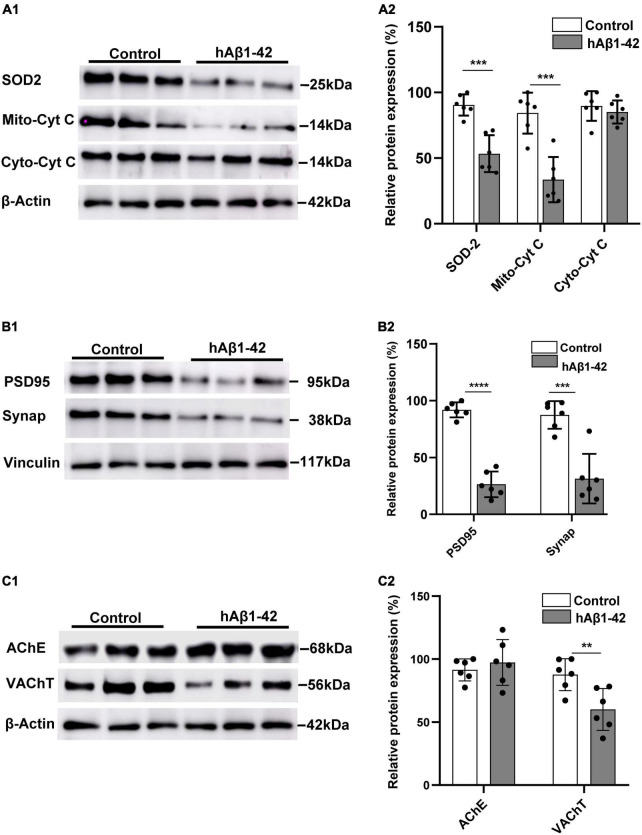
Effects of hAβ_1–42_ on key mitochondrial elements, synaptic proteins, and cholinergic markers in the entorhinal cortex. **(A1)** Representative immunoblots of mitochondrial superoxide dismutase 2 (SOD2), mitochondrial cytochrome c (Mito-cyt C), cytosolic cytochrome c (Cyto-cyt C), and β-actin loading control in entorhinal lysates treated with hAβ_1–42_ and control medium. **(A2)** Normalized expression of SOD2, Mito-cyt C, and Cyto-cyt C in hAβ_1–42_-treated entorhinal samples compared to control (*n* = 6). **(B1)** Representative immunoblots of postsynaptic density protein (PSD95), presynaptic marker synaptophysin (Synap), and vinculin loading control in entorhinal lysates. **(B2)** Quantification data showing the normalized expression of both PSD95 and Synap in slices incubated with hAβ_1–42_ vs. control (*n* = 6). **(C1)** Representative immunoblots of cholinergic markers acetylcholinesterase (AChE), vesicular acetylcholine transporter (VAChT), and β-actin (loading control). **(C)** Bar graphs showing normalized expression of AChE and VAChT in hAβ_1–42_–treated slices and control (*n* = 6) (**p < 0.01; ***p < 0.005; ****p < 0.0001).

Our finding that hAβ_1–42_ rapidly impairs mitochondrial respiration in the EC suggests that a resulting oxidative redox imbalance, due to increased leakage of mitochondrial electrons and ROS formation, could have deleterious effects on the mitochondrial antioxidant system. We therefore characterized the expression of the mitochondrial oxidative scavenger enzyme SOD2, and both mitochondrial and cytosolic cytochrome c protein. Representative immunoblots and quantification data show that, compared to control, hAβ_1–42_ markedly reduced the relative expression of SOD2 (53.3 ± 14.0 vs. 90.4 ± 8.1 in controls; *p* = 0.0002) (Figure 2A). Mitochondrial cytochrome c expression was also reduced by application of hAβ_1–42_ (33.6 ± 17.2 vs. 86.0 ± 12.3 in controls; *p* = 0.0001), and this effect was specific to mitochondria because immunoexpression of cytosolic cytochrome c was not significantly affected (81.7 ± 13.6 vs. 89.6 ± 11.3 *p* = 0.298).

A reduction in the efficiency and energy production capacity of the mitochondrial electron transport chain, increased mitochondrial ROS, and oxidative stress could impact both pre- and post-synaptic elements, and we therefore evaluated the effects of hAβ_1–42_ on the presynaptic marker synaptophysin, and the postsynaptic density protein 95 (PSD95). We found that hAβ_1–42_-induced mitochondrial dysregulation was accompanied by a marked reduction in relative immunoexpression of both PSD95 (91.9 ± 6.6 and 26.4 ± 11.3; *p* < 0.0001) and synaptophysin (87.5 ± 12.2 and 31.3 ± 21.8; *p* = 0.0003) (Figure 2B).

Reductions in cholinergic function in the EC is one of the earliest manifestations of AD (Hampel et al., 2018), and we therefore assessed the effects of hAβ_1–42_ on the expression of the vesicular acetylcholine transporter (VAChT) and the degradative enzyme acetylcholinesterase (AChE). Although hAβ_1–42_ did not cause a significant change in AChE immunoexpression when compared to control (97.4 ± 18.2 vs. 91.5 ± 8.7 in controls; *p* = 0.5433), hAβ_1–42_ markedly reduced the expression of VAChT in entorhinal lysates vs. control (60.0 ± 16.6 vs. 87.7 ± 12.7; *p* = 0.0312) (Figure 2C), suggesting a disruption in the function of cholinergic terminals in the EC.

### Mitochondria-targeted antioxidant mitoquinone mesylate and EUK134 reduce hAβ_1–42_-induced impairments in synaptic proteins

**FIGURE 3:**
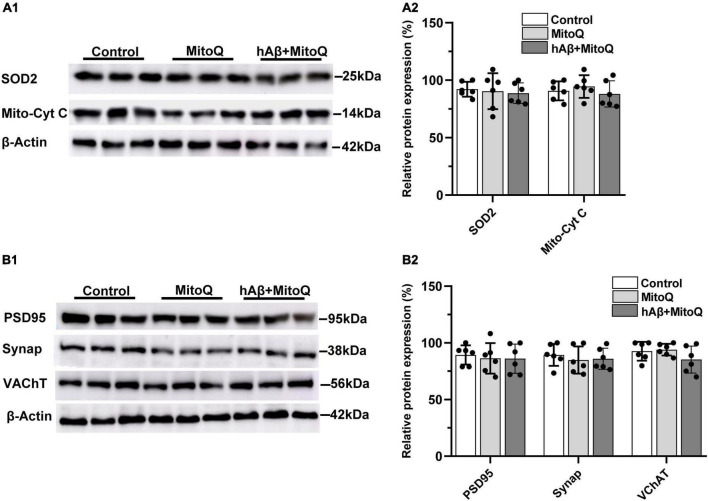
Inhibiting increases in reactive oxygen species with MitoQ prevents hAβ_1–42_-induced changes in mitochondrial and synaptic proteins. **(A1)** Representative immunoblots of superoxide dismutase 2 (SOD2), mitochondrial cytochrome c (Mito-cyt C), and the loading control β-Actin, are shown in slices treated with MitoQ, hAβ_1–42_ with MitoQ, and control. **(A2)** Normalized relative expression of SOD2 and Mito-cyt C proteins (*n* = 6). **(B1)** Representative immunoblots of postsynaptic density protein PSD95, presynaptic marker synaptophysin (Synap.), vesicular acetylcholine transporter (VAChT), and the loading control β-Actin are shown in tissue treated with MitoQ, hAβ_1–42_ with MitoQ, and control. Bar graphs indicate normalized relative expression of PSD95, Synap., and VAChT **(B2)**.

To determine if increased ROS associated with mitochondrial dysregulation is related to the reductions in mitochondrial enzymes and synaptic proteins induced by hAβ_1–42_, we tested the ability of two novel, structurally distinct ROS scavengers (MitoQ and EUK134) to block protein reductions induced by hAβ_1–42_. The mitochondria-targeted antioxidant MitoQ administered alone had no significant effect on the expression of mitochondrial enzymes, but was found to block changes in both SOD2 and mitochondrial cytochrome c induced by hAβ_1–42_ (compare Figure 3A and Figure 2A). There was no significant main effect of treatment on the relative expression of SOD2 between control slices (92.2 ± 6.4) and slices incubated with MitoQ alone (90.4 ± 15.6) or MitoQ with hAβ_1–42_ (88.7 ± 8.9) [*F*_(2_, _15)_ = 0.15, *p* = 0.8646]. Similarly, the expression of mitochondrial cytochrome c protein was not significantly different between control slices (90.7.0 ± 8.3) and slices treated with either MitoQ (94.5 ± 9.8) or MitoQ and hAβ_1–42_ (88.0 ± 11.4) [*F*_(2_, _15)_ = 0.65, *p* = 0.5355] (Figure 3A).

We then assessed if blocking excess ROS induced by hAβ_1–42_ using MitoQ would also block reductions in the immunoexpression of PSD95, synaptophysin, and the vesicular acetylcholine transporter (VChAT). The application of MitoQ had no effect alone, but it prevented reductions in all three proteins induced by hAβ_1–42_ (compare Figure 3B and Figures 2B,C). There were no significant main effects of treatment (*n* = 6) on the expression of PSD95 [*F*_(2_, _15)_ = 0.14, *p* = 0.8742], synaptophysin [*F*_(2_, _15)_ = 0.31, *p* = 0.7386], and VChAT [*F*_(2_, _15)_ = 1.66, *p* = 0.2226]. Preventing increases in ROS induced by hAβ_1–42_ can therefore prevent degradation of key synaptic proteins.

**FIGURE 4:**
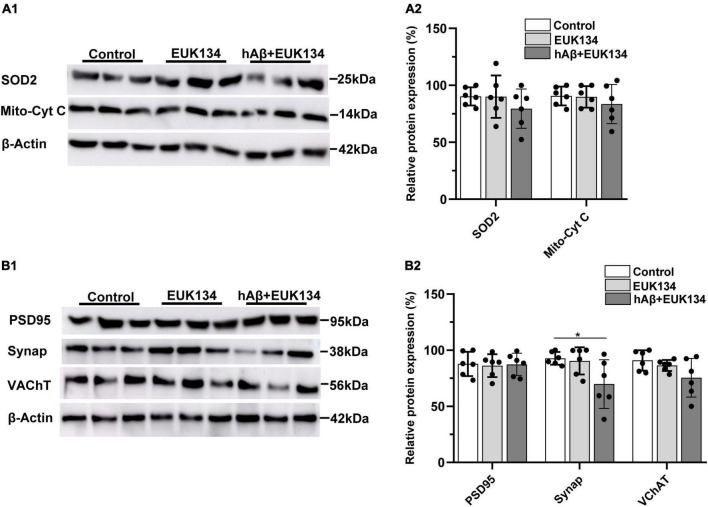
Inhibiting increases in reactive oxygen species with EUK134 prevents hAβ_1–42_-induced changes in mitochondrial and synaptic proteins. **(A1)** Representative immunoblots of superoxide dismutase 2 (SOD2), mitochondrial cytochrome c (Mito-cyt C), and the loading control β-Actin, are shown in slices treated with EUK134, hAβ_1–42_ with EUK134, and control. **(A2)** Normalized relative expression of SOD2 and Mito-cyt C proteins (*n* = 6). **(B1)** Representative immunoblots of postsynaptic density protein PSD95, presynaptic marker synaptophysin (Synap.), vesicular acetylcholine transporter (VAChT), and the loading control β-Actin are shown for tissue treated with EUK134, hAβ_1–42_ with EUK134, and control. Bar graphs indicate normalized relative expression of PSD95, Synap., and VAChT **(B2)** (*p < 0.05).

The idea that increases in mitochondrial ROS induced by hAβ_1–42_ mediate synaptic degeneration in entorhinal slices was further tested by determining if the synthetic SOD and catalase mimetic EUK134 could block the reductions in synaptic and mitochondrial proteins induced by hAβ_1–42_. Application of EUK134 blocked reductions in both SOD2 and mitochondrial cytochrome c induced by hAβ_1–42_ (compare Figure 4A and Figure 2A), and there were no significant main effects of treatment with EUK134, or with EUK134 and hAβ_1–42_, (*n* = 6), on the relative expression of SOD2 [*F*_(2_, _15)_ = 0.95, *p* = 0.4084] or mitochondrial cytochrome c [*F*_(2_, _15)_ = 0.61, *p* = 0.5578].

We also found that EUK134 rescued hAβ_1–42_-induced reductions in immunoexpression of PSD95 and VChAT, and partially rescued hAβ_1–42_-induced reductions in the presynaptic protein synaptophysin (compare Figure 4B and Figures 2B,C). There was no significant effect of treatment with EUK134 or EUK134 and hAβ_1–42_ on the relative expression of PSD95 [*F*_(2_, _15)_ = 0.04, *p* = 0.9650] and VChAT [*F*_(2_, _15)_ = 2.83, *p* = 0.0905]. There was, however, a significant main effect of treatment on synaptophysin immunoexpression [*F*_(2_, _15)_ = 4.44, *p* = 0.0305] in which the expression of synaptophysin was significantly reduced in slices treated with EUK134 + hAβ_1–42_ relative to control slices (69.8 ± 21.6 vs. 92.7 ± 5.7 in controls; *p* = 0.0480), but not relative to slices treated with EUK134 alone (90.4 ± 12.1; *p* = 0.0811). The mean expression of synaptophysin following treatment with hAβ_1–42_ in the presence of EUK134 (69.8 ± 21.6) was greater than that following treatment with hAβ_1–42_ alone (31.3 ± 21.8; Figure 2B), however, suggesting that EUK134 was partially protective in preventing reductions in synaptophysin. These findings suggest that hAβ_1–42_-induced rapid synaptic impairments in entorhinal tissue can be significantly rescued by inhibiting mitochondrial ROS levels.

## Discussion

A previous report from our lab has shown that hAβ_1–42_ causes rapid degeneration of presynaptic and postsynaptic elements in the EC through activation of GluN2A- and GluN2B-containing NMDA glutamate receptors (Olajide and Chapman, 2021). The selective vulnerability of the EC to early AD-related synaptic damage and neurodegeneration (Du et al., 2004; Velayudhan et al., 2013; Zhou et al., 2016), may reflect the dysregulation of neural mechanisms including NMDA glutamate receptors that contribute to learning and memory (Olajide et al., 2021). Aβ can accumulate in mitochondria where it impairs mitochondrial dynamics and upregulates oxidative stress by impairing mitochondrial respiratory function and the production of adenosine triphosphate (ATP) (Wang et al., 2014, 2020; Ashleigh et al., 2022). Further, activation of NMDA receptors by hAβ_1–42_ can lead to increases in calcium influx, the rapid facilitation of AMPA glutamate receptor responses, and hyperexcitability that increases metabolic demands on mitochondria, ultimately culminating in oxidative stress and synaptic dysfunction (Findley et al., 2019; Guo et al., 2020; Wang et al., 2020). We have found here that exposure to 1 μM hAβ_1–42_ for a period of 3 h markedly reduces mitochondrial respiratory function and integrity in the EC. Further, the resulting increase in ROS appears to be a major driver of the rapid degeneration of both pre- and post-synaptic proteins in the EC because the ROS scavengers Mito-Q and EUK134 strongly inhibited reductions in synaptophysin and PSD-95. The reductions in synaptic proteins observed here imply a reduction in number of synapses, but it is not clear if this would be expressed following this relatively brief application of hAβ_1–42_. However, these results point to a major role of mitochondrial dysregulation in the synaptic degeneration induced by hAβ_1–42_, which may contribute to the early progression of AD in the EC (Olajide et al., 2021). The present results may reflect mitochondrial dysfunction in glia as well as neurons, and mitochondrial dysfunction in glia is also thought to be a major contributor to the progression of AD (Mulica et al., 2021).

In our experimental model we have applied moderate concentrations of Aβ to wildtype slices for 3 h, but the progression of AD involves circulation of lower concentrations of Aβ over greatly extended periods of time. Although our model may reflect degenerative mechanisms that are expressed early in the progression of AD, and reflect the early selective vulnerability of the EC to Aβ toxicity (Olajide et al., 2021), the changes in mitochondrial function and synaptic proteins that we have observed might also occur in later stages of AD after prolonged exposure to lower concentrations of Aβ.

### Human Aβ_1–42_ impairs mitochondrial respiration and function in the entorhinal cortex

We have found here that hAβ_1–42_ markedly reduces mitochondrial-coupled oxygen consumption, and functional membrane integrity as reflected by oxygen use in the presence of cytochrome c. High-resolution respirometry showed no significant changes in non-phosphorylating LEAK respiration during the addition of the Complex I-linked substrates malate, pyruvate or glutamate, but the OXPHOS capacity of Complex I-linked activity was markedly reduced by hAβ_1–42_, as shown by the approximately fivefold reduction in oxygen utilization during the addition of ADP. Exposure to hAβ_1–42_ also impaired the function of Complex II, because the addition of succinate, which reflects the combined OXPHOS capacity of Complex I and Complex II, was also significantly reduced. The rapid defects in mitochondrial-related metabolic pathways provide direct evidence that impaired bioenergetic machinery in mitochondria is an early driver of AD-related pathology in the EC. Similarly, OXPHOS and associated pathways are significantly downregulated within the hippocampus during AD, and reductions in Complex I, III, and IV of OXPHOS have been reported in both early and definite AD brains (Manczak et al., 2004; Brooks et al., 2007).

Further, the indication that hAβ_1–42_-induced impairments in entorhinal electron transport chain complexes can dysregulate bioenergetics is corroborated by our findings that the efficiency of the link between oxidation and the production of ATP by phosphorylation of ADP was reduced by hAβ_1–42_, as reflected by both reductions in the uncoupling capacity of the mitochondria during the addition of FCCP, and by a reduction in the ACR. The ACR expresses the rate of oxygen consumption during ADP phosphorylation relative to the rate of non-phosphorylating LEAK-respiration induced by glutamate. Our data corresponds with several reports indicating a large and consistent decline in mitochondrial substrate utilization and energy production in the neocortex during the prodromal stages of AD (Croteau et al., 2018; Wang et al., 2020), suggesting that mitochondrial bioenergetics dysfunction plays an early role in the pathogenesis of AD in the EC.

Mitochondrial cytochrome c transfers electrons to complex IV in the electron transport chain which is the primary site of cellular oxygen consumption (Timón-Gómez et al., 2018). We found that hAβ_1–42_ caused about a 2.5-fold reduction in mitochondrial cytochrome c function, suggesting alterations to the mitochondrial membrane in entorhinal tissue. We further found that, although total cytoplasmic cytochrome c protein expression was unaffected, the expression of mitochondrial cytochrome c was greatly reduced by treatment with hAβ_1–42_. An increase in cytosolic cytochrome c could be expected from previous work showing that Aβ can activate intrinsic apoptotic pathways and the release of mitochondrial cytochrome c into the cytosol (Kim et al., 2014), but the lack of an increase in cytosolic cytochrome c in the present study may be due to the relatively brief duration of exposure to Aβ. Deficiency of cytochrome c oxidase (Complex IV) is the most common defect in the mitochondrial electron transport chain in AD, leading to an increase in ROS production, a decrease in energy stores, and a disruption of energy metabolism (Rak et al., 2016). We found a marked reduction in the expression of SOD2, a key antioxidant enzyme that scavenges superoxide in mitochondria, and this is consistent with a rapid increase in mitochondrial ROS production induced by hAβ_1–42_ that depletes the energy metabolism machinery in the EC.

Although hAβ_1–42_ significantly reduced respiration and bioenergetics, it did not alter the expression of protein markers for the five mitochondrial Complexes or the expression of the mitochondrial voltage-dependent anion channel 1 (VDAC1). VDAC1 is the most abundant protein on the outer membrane of mitochondria, and it serves as a gatekeeper for the passage of metabolites, and is crucial for the metabolic functions of mitochondria (Camara et al., 2017). It is well established that the expression of mitochondrial proteins and genes are among the most prominent changes in the cortex during AD (Cottrell et al., 2001; Valla et al., 2001; Minjarez et al., 2016; Adav et al., 2019), and the lack of a significant effect of hAβ_1–42_ on the expression of mitochondrial complex proteins observed here may reflect the resistance of these proteins to rapid degradation over the relativity short incubation time.

### Preventing increases in reactive oxygen species inhibits hAβ_1–42_-induced synaptic changes

Reductions in synaptic proteins induced by hAβ_1–42_ are likely to have resulted from increased ROS and oxidative stress in entorhinal neurons. Incubation in hAβ_1–42_ resulted in a marked reduction in SOD2, which is the primary antioxidant enzyme that scavenges superoxide in mitochondria, suggesting that SOD2 may have been depleted due to increases in ROS and overwhelming activity-dependent utilization of SOD2. The reduction in SOD2 may have further impaired ROS scavenging in entorhinal neurons thereby exacerbating oxidative damage and a loss of mitochondrial cytochrome c function. Excessive ROS and oxidative stress have been extensively linked to synaptic loss associated with aging and AD (Patten et al., 2010; Tönnies and Trushina, 2017; Guo et al., 2020), and increases in ROS associated with mitochondrial dysfunction that exceed the scavenging ability of entorhinal neurons is likely to have contributed to the observed loss of synaptic proteins. Consistent with our previous findings in the EC (Olajide and Chapman, 2021), both the presynaptic marker synaptophysin and the postsynaptic marker PSD-95, were reduced by hAβ_1–42_.

The degeneration of cholinergic terminals in the EC is one of the earliest neuropathologies in AD (Francis et al., 1999; Gil-Bea et al., 2005; Hamam et al., 2007; Schaeffer and Gattaz, 2008). Although we found that the degradative enzyme acetylcholinesterase was not affected by hAβ_1–42_, we did observe a reduction in the vesicular acetylcholine transporter (VAChT), suggesting a reduced function in cholinergic terminals. Several other reports have also shown marked reductions in the expression of VAChT following Aβ infusion in rats and in AD models (Ikeda et al., 2000; Pákáski and Kálmán, 2008; Schliebs and Arendt, 2011; Hampel et al., 2018).

ROS-mediated oxidative stress is thought to have a major role in synaptic dysregulation and neurodegeneration in AD, and various antioxidants, and the genetic overexpression of SOD2, have been explored as therapeutic strategies for AD (Dumont et al., 2009; Massaad et al., 2009; Bonda et al., 2010; Olajide et al., 2017a,b, 2018; Tönnies and Trushina, 2017; Misrani et al., 2021). We co-incubated slices with hAβ_1–42_ and the ROS inhibitors MitoQ or EUK134 to determine if reducing oxidative stress could prevent the degeneration of synaptic proteins in the EC. Both MitoQ and EUK134 inhibited hAβ_1–42_-induced reductions in both SOD2 and mitochondrial cytochrome c, likely by maintaining a sufficient ROS scavenging capacity in entorhinal neurons and preventing overwhelming demands on SOD2. In addition, co-incubation with MitoQ blocked hAβ_1–42_-induced decreases in synaptophysin, PSD-95, and VAChT, indicating that oxidative stress arising from insufficient mitochondrial capacity can drive degeneration of these synaptic proteins. Similarly, the ROS inhibitor EUK134 prevented reductions in PSD-95 and VAChT, and greatly attenuated reductions in synaptophysin induced by hAβ_1–42_. Others have also found that decreasing ROS using MitoQ and EUK134 can prevent synaptic degeneration induced by oxidative stress in cortical and hippocampal neurons (Ma et al., 2011; Mcmanus et al., 2011; Reddy et al., 2012; Sanmartín et al., 2017; Yu et al., 2018; Cenini and Voos, 2019). These studies suggest that the protective effect of ROS inhibition that we have observed following acute application of hAβ_1–42_ to wildtype slices might be similarly protective for the advancement of neurodegeneration in more chronic models of AD.

## Conclusion

Mitochondrial dysfunction resulting in increased ROS production and oxidative stress precedes the formation of neuritic plaques and neurofibrillary tangles, and is thought to contribute substantially to the earliest stages of AD and the onset of cognitive decline and memory loss (Uttara et al., 2009; Wang et al., 2014; Tönnies and Trushina, 2017; Ashleigh et al., 2022). We have shown here that short-term exposure of wild-type EC slices to hAβ_1–42_ results in deficits in mitochondrial respiration, and have provided evidence that oxidative stress associated with increased ROS is a major factor in the rapid degeneration of key pre- and post-synaptic proteins in the EC. Further, these mechanisms may be a major contributor to the reduction in cholinergic transmission in the EC, which is an early pathology thought to contribute to cognitive decline.

## Data availability statement

The raw data supporting the conclusions of this article will be made available by the authors, without undue reservation.

## Ethics statement

The animal study was reviewed and approved by Animal Research Ethics Committee, Concordia University.

## Author contributions

OO, AB, and CC contributed to conception and design of the study. OO, AB, and CL acquired and analyzed the data. OO wrote the first draft of the manuscript. All authors contributed to manuscript revision and approved the submitted version.

## References

[B1] Adav S. S. Park J. E. Sze S. K. (2019). Quantitative profiling brain proteomes revealed mitochondrial dysfunction in Alzheimer’s disease. *Mol. Brain* 12:8. 10.1186/S13041-019-0430-Y/FIGURES/6 30691479 PMC6350377

[B2] Akhter F. Chen D. Yan S. F. Yan S. S. (2017). Mitochondrial perturbation in Alzheimer’s disease and diabetes. *Prog. Mol. Biol. Transl. Sci.* 146 341–361. 10.1016/bs.pmbts.2016.12.019 28253990 PMC5896572

[B3] Armand-Ugon M. Ansoleaga B. Berjaoui S. Ferrer I. (2017). Reduced mitochondrial activity is early and steady in the entorhinal cortex but it is mainly unmodified in the frontal cortex in Alzheimer’s disease. *Curr. Alzheimer Res.* 14 1327–1334. 10.2174/1567205014666170505095921 28474567

[B4] Arendt T. (2009). Synaptic degeneration in Alzheimer’s disease. *Acta Neuropathol.* 118 167–179. 10.1007/s00401-009-0536-x 19390859

[B5] Ashleigh T. Swerdlow R. H. Beal M. F. (2022). The role of mitochondrial dysfunction in Alzheimer’s disease pathogenesis. *Alzheimers Dement.* 10.1002/alz.12683 35522844

[B6] Bitan G. Vollers S. S. Teplow D. B. (2003). Elucidation of primary structure elements controlling early amyloid β-protein oligomerization. *J. Biol. Chem.* 278 34882–34889. 10.1074/jbc.M300825200 12840029

[B7] Bonda D. J. Wang X. Perry G. Nunomura A. Tabaton M. Zhu X. (2010). Oxidative stress in Alzheimer disease: A possibility for prevention. *Neuropharmacology* 59 290–294. 10.1016/j.neuropharm.2010.04.005 20394761

[B8] Brooks W. M. Lynch P. J. Ingle C. C. Hatton A. Emson P. C. Faull R. L. M. (2007). Gene expression profiles of metabolic enzyme transcripts in Alzheimer’s disease. *Brain Res.* 1127 127–135. 10.1016/J.BRAINRES.2006.09.106 17109828

[B9] Calkins M. J. Manczak M. Mao P. Shirendeb U. Reddy P. H. (2011). Impaired mitochondrial biogenesis, defective axonal transport of mitochondria, abnormal mitochondrial dynamics and synaptic degeneration in a mouse model of Alzheimer’s disease. *Hum. Mol. Genet.* 20 4515–4529. 10.1093/hmg/ddr381 21873260 PMC3209824

[B10] Camara A. K. S. Zhou Y. F. Wen P. C. Tajkhorshid E. Kwok W. M. (2017). Mitochondrial VDAC1: A key gatekeeper as potential therapeutic target. *Front. Physiol.* 8:460. 10.3389/FPHYS.2017.00460/BIBTEX 28713289 PMC5491678

[B11] Cavallucci V. Ferraina C. D’Amelio M. (2013). Key role of mitochondria in Alzheimer’s disease synaptic dysfunction. *Curr. Pharm. Des.* 19 6440–6450. 10.2174/1381612811319360005 23432718

[B12] Cenini G. Voos W. (2019). Mitochondria as potential targets in Alzheimer disease therapy: An update. *Front. Pharmacol.* 10:902. 10.3389/FPHAR.2019.00902/BIBTEX 31507410 PMC6716473

[B13] Coleman P. D. Yao P. J. (2003). Synaptic slaughter in Alzheimer’s disease. *Neurobiol. Aging* 24 1023–1027. 10.1016/j.neurobiolaging.2003.09.001 14643374

[B14] Cottrell D. A. Blakely E. L. Johnson M. A. Ince P. G. Turnbull D. M. (2001). Mitochondrial enzyme-deficient hippocampal neurons and choroidal cells in AD. *Neurology* 57 260–264. 10.1212/WNL.57.2.260 11468310

[B15] Croteau E. Castellano C. A. Fortier M. Bocti C. Fulop T. Paquet N. (2018). A cross-sectional comparison of brain glucose and ketone metabolism in cognitively healthy older adults, mild cognitive impairment and early Alzheimer’s disease. *Exp. Gerontol.* 107 18–26. 10.1016/J.EXGER.2017.07.004 28709938

[B16] Du A. T. Schuff N. Kramer J. H. Ganzer S. Zhu X. P. Jagust W. J. (2004). Higher atrophy rate of entorhinal cortex than *Hippocampus* in AD. *Neurology* 62 422–427. 10.1212/01.WNL.0000106462.72282.90 14872024 PMC1820859

[B17] Dumont M. Wille E. Stack C. Calingasan N. Y. Beal M. F. Lin M. T. (2009). Reduction of oxidative stress, amyloid deposition, and memory deficit by manganese superoxide dismutase overexpression in a transgenic mouse model of Alzheimer’s disease. *FASEB J.* 23 2459–2466. 10.1096/FJ.09-132928 19346295 PMC2717785

[B18] Esposito L. Raber J. Kekonius L. Yan F. Yu G. Q. Bien-Ly N. (2006). Reduction in mitochondrial superoxide dismutase modulates Alzheimer’s disease-like pathology and accelerates the onset of behavioral changes in human amyloid precursor protein transgenic mice. *J. Neurosci.* 26 5167–5179. 10.1523/JNEUROSCI.0482-06.2006 16687508 PMC6674260

[B19] Fan L. Mao C. Hu X. Zhang S. Yang Z. Hu Z. (2020). New insights into the pathogenesis of Alzheimer’s disease. *Front. Neurol.* 10:1312. 10.3389/fneur.2019.01312 31998208 PMC6965067

[B20] Findley C. A. Bartke A. Hascup K. N. Hascup E. R. (2019). Amyloid beta-related alterations to glutamate signaling dynamics during Alzheimer’s disease progression. *ASN Neuro* 11:1759091419855541. 10.1177/1759091419855541 31213067 PMC6582288

[B21] Francis P. T. Palmer A. M. Snape M. Wilcock G. K. (1999). The cholinergic hypothesis of Alzheimer’s disease: A review of progress. *J. Neurol. Neurosurg. Psychiatry* 66 137–147. 10.1136/jnnp.66.2.137 10071091 PMC1736202

[B22] Gil-Bea F. J. García-Alloza M. Domínguez J. Marcos B. Ramírez M. J. (2005). Evaluation of cholinergic markers in Alzheimer’s disease and in a model of cholinergic deficit. *Neurosci. Lett.* 375 37–41. 10.1016/j.neulet.2004.10.062 15664119

[B23] Glovaci I. Chapman C. A. (2019). Dopamine induces release of calcium from internal stores in layer II lateral entorhinal cortex fan cells. *Cell Calcium* 80 103–111. 10.1016/j.ceca.2019.04.003 30999216

[B24] Grubman A. Chew G. Ouyang J. F. Sun G. Choo X. Y. McLean C. (2019). A single-cell atlas of entorhinal cortex from individuals with Alzheimer’s disease reveals cell-type-specific gene expression regulation. *Nat. Neurosci.* 22 2087–2097. 10.1038/s41593-019-0539-4 31768052

[B25] Guo T. Zhang D. Zeng Y. Huang T. Y. Xu H. Zhao Y. (2020). Molecular and cellular mechanisms underlying the pathogenesis of Alzheimer’s disease. *Mol. Neurodegener.* 15:40. 10.1186/s13024-020-00391-7 32677986 PMC7364557

[B26] Haass C. Selkoe D. J. (2007). Soluble protein oligomers in neurodegeneration: Lessons from the Alzheimer’s amyloid β-peptide. *Nat. Rev. Mol. Cell Biol.* 8 101–112. 10.1038/nrm2101 17245412

[B27] Hamam B. N. Sinai M. Poirier G. Chapman C. A. (2007). Cholinergic suppression of excitatory synaptic responses in layer II of the medial entorhinal cortex. *Hippocampus* 17 103–113. 10.1002/hipo.20249 17146776

[B28] Hampel H. Hardy J. Blennow K. Chen C. Perry G. Kim S. H. (2021). The amyloid-β pathway in Alzheimer’s disease. *Mol. Psychiatry* 26 5481–5503. 10.1038/s41380-021-01249-0 34456336 PMC8758495

[B29] Hampel H. Mesulam M. M. Cuello A. C. Farlow M. R. Giacobini E. Grossberg G. T. (2018). The cholinergic system in the pathophysiology and treatment of Alzheimer’s disease. *Brain* 141 1917–1933. 10.1093/brain/awy132 29850777 PMC6022632

[B30] Ikeda E. Shiba K. Mori H. Ichikawa A. Sumiya H. Kuji I. (2000). Reduction of vesicular acetylcholine transporter in beta-amyloid protein-infused rats with memory impairment. *Nucl. Med. Commun.* 21 933–937. 10.1097/00006231-200010000-00007 11130334

[B31] Ionescu-Tucker A. Cotman C. W. (2021). Emerging roles of oxidative stress in brain aging and Alzheimer’s disease. *Neurobiol. Aging* 107 86–95. 10.1016/J.NEUROBIOLAGING.2021.07.014 34416493

[B32] Khan U. A. Liu L. Provenzano F. A. Berman D. E. Profaci C. P. Sloan R. (2014). Molecular drivers and cortical spread of lateral entorhinal cortex dysfunction in preclinical Alzheimer’s disease. *Nat. Neurosci.* 17 304–311. 10.1038/nn.3606 24362760 PMC4044925

[B33] Khosravi S. Harner M. E. (2020). The MICOS complex, a structural element of mitochondria with versatile functions. *Biol. Chem.* 401 765–778. 10.1515/hsz-2020-0103 32229686

[B34] Kim J. Yang Y. Song S. S. Na J. H. Oh K. J. Jeong C. (2014). Beta-amyloid oligomers activate apoptotic BAK pore for cytochrome c release. *Biophys. J.* 107 1601–1608. 10.1016/j.bpj.2014.07.074 25296312 PMC4190645

[B35] Li F. Calingasan N. Y. Yu F. Mauck W. M. Toidze M. Almeida C. G. (2004). Increased plaque burden in brains of APP mutant MnSOD heterozygous knockout mice. *J. Neurochem.* 89 1308–1312. 10.1111/J.1471-4159.2004.02455.X 15147524

[B36] Ma T. Hoeffer C. A. Wong H. Massaad C. A. Zhou P. Iadecola C. (2011). Amyloid β-induced impairments in hippocampal synaptic plasticity are rescued by decreasing mitochondrial superoxide. *J. Neurosci.* 31 5589–5595. 10.1523/JNEUROSCI.6566-10.2011 21490199 PMC3095121

[B37] Manczak M. Park B. S. Jung Y. Reddy P. H. (2004). Differential expression of oxidative phosphorylation genes in patients with Alzheimer’s disease: Implications for early mitochondrial dysfunction and oxidative damage. *Neuromolecular Med.* 5 147–162. 10.1385/NMM 15075441

[B38] Marsh J. Alifragis P. (2018). Synaptic dysfunction in Alzheimer’s disease: The effects of amyloid beta on synaptic vesicle dynamics as a novel target for therapeutic intervention. *Neural Regen. Res.* 13 616–623. 10.4103/1673-5374.230276 29722304 PMC5950662

[B39] Massaad C. A. Washington T. M. Pautler R. G. Klann E. (2009). Overexpression of SOD-2 reduces hippocampal superoxide and prevents memory deficits in a mouse model of Alzheimer’s disease. *Proc. Natl. Acad. Sci. U.S.A.* 106:13576. 10.1073/PNAS.0902714106 19666610 PMC2726352

[B40] Masters C. L. Selkoe D. J. (2012). Biochemistry of amyloid β-protein and amyloid deposits in Alzheimer disease. *Cold Spring Harb. Perspect. Med.* 2:a006262. 10.1101/cshperspect.a006262 22675658 PMC3367542

[B41] Mcmanus M. J. Murphy M. P. Franklin J. L. (2011). The mitochondria-targeted antioxidant mitoq prevents loss of spatial memory retention and early neuropathology in a transgenic mouse model of Alzheimer’s disease. *J. Neurosci.* 31:15703. 10.1523/JNEUROSCI.0552-11.2011 22049413 PMC3334845

[B42] Minjarez B. Calderón-González K. G. Rustarazo M. L. V. Herrera-Aguirre M. E. Labra-Barrios M. L. Rincon-Limas D. E. (2016). Identification of proteins that are differentially expressed in brains with Alzheimer’s disease using iTRAQ labeling and tandem mass spectrometry. *J. Proteom.* 139 103–121. 10.1016/J.JPROT.2016.03.022 27012543

[B43] Misrani A. Tabassum S. Yang L. (2021). Mitochondrial dysfunction and oxidative stress in Alzheimer’s disease. *Front. Aging Neurosci.* 13:57. 10.3389/FNAGI.2021.617588/BIBTEX PMC7930231 33679375

[B44] Mucke L. Selkoe D. J. (2012). Neurotoxicity of amyloid β-protein: Synaptic and network dysfunction. *Cold Spring Harb. Perspect. Med.* 2:a006262. 10.1101/cshperspect.a006338 22762015 PMC3385944

[B45] Mulica P. Grünewald A. Pereira S. L. (2021). Astrocyte-neuron metabolic crosstalk in neurodegeneration: A mitochondrial perspective. *Front. Endocrinol.* 12:668517. 10.3389/fendo.2021.668517 34025580 PMC8138625

[B46] Murphy M. P. (2009). How mitochondria produce reactive oxygen species. *Biochem J.* 417 1–13. 10.1042/BJ20081386 19061483 PMC2605959

[B47] Nunomura A. Perry G. Aliev G. Hirai K. Takeda A. Balraj E. K. (2001). Oxidative damage is the earliest event in Alzheimer disease. *J. Neuropathol. Exp. Neurol.* 60 759–767. 10.1093/JNEN/60.8.759 11487050

[B48] Olajide O. J. Chapman C. A. (2021). Amyloid-β (1-42) peptide induces rapid NMDA receptor-dependent alterations at glutamatergic synapses in the entorhinal cortex. *Neurobiol. Aging* 105 296–309. 10.1016/J.NEUROBIOLAGING.2021.05.006 34144329

[B49] Olajide O. J. Asogwa N. T. Moses B. O. Oyegbola C. B. (2017a). Multidirectional inhibition of cortico-hippocampal neurodegeneration by kolaviron treatment in rats. *Metab. Brain Dis.* 32 1147–1161. 10.1007/s11011-017-0012-6 28405779

[B50] Olajide O. J. Fatoye J. O. Idowu O. F. Ilekoya D. Gbadamosi I. T. Gbadamosi M. T. (2018). Reversal of behavioral decline and neuropathology by a complex vitamin supplement involves modulation of key neurochemical stressors. *Environ. Toxicol. Pharmacol.* 62 120–131. 10.1016/j.etap.2018.07.005 30005307

[B51] Olajide O. J. Suvanto M. E. Chapman C. A. (2021). Molecular mechanisms of neurodegeneration in the entorhinal cortex that underlie its selective vulnerability during the pathogenesis of Alzheimer’s disease. *Biol. Open* 10:bio056796. 10.1242/bio.056796 33495355 PMC7860115

[B52] Olajide O. J. Yawson E. O. Gbadamosi I. T. Arogundade T. T. Lambe E. Obasi K. (2017b). Ascorbic acid ameliorates behavioural deficits and neuropathological alterations in rat model of Alzheimer’s disease. *Environ. Toxicol. Pharmacol.* 50 200–211. 10.1016/j.etap.2017.02.010 28192749

[B53] O’Nuallain B. Williams A. D. Westermark P. Wetzel R. (2004). Seeding specificity in amyloid growth induced by heterologous fibrils. *J. Biol. Chem.* 279 17490–17499. 10.1074/jbc.M311300200 14752113

[B54] Overk C. R. Masliah E. (2014). Pathogenesis of synaptic degeneration in Alzheimer’s disease and Lewy body disease. *Biochem. Pharmacol.* 88 508–516. 10.1016/j.bcp.2014.01.015 24462903 PMC3973539

[B55] Pákáski M. Kálmán J. (2008). Interactions between the amyloid and cholinergic mechanisms in Alzheimer’s disease. *Neurochem. Int.* 53 103–111. 10.1016/j.neuint.2008.06.005 18602955

[B56] Patten D. A. Germain M. Kelly M. A. Slack R. S. (2010). Reactive oxygen species: Stuck in the middle of neurodegeneration. *J. Alzheimers Dis.* 20 S357–S367. 10.3233/JAD-2010-100498 20421690

[B57] Paxinos G. Watson C. (1997). *The rat brain in stereotaxic coordinates*, 3rd Edn. San Diego, CA: Academic Press.

[B58] Pickett E. K. Rose J. McCrory C. McKenzie C. A. King D. Smith C. (2018). Region-specific depletion of synaptic mitochondria in the brains of patients with Alzheimer’s disease. *Acta Neuropathol.* 136 747–757. 10.1007/s00401-018-1903-2 30191401 PMC6208730

[B59] Rak M. Bénit P. P. Chrétien D. Bouchereau J. Schiff M. El-Khoury R. (2016). Mitochondrial cytochrome c oxidase deficiency. *Clin. Sci.* 130 393–407. 10.1042/CS20150707 26846578 PMC4948581

[B60] Reddy P. H. Tripathi R. Troung Q. Tirumala K. Reddy T. P. Anekonda V. (2012). abnormal mitochondrial dynamics and synaptic degeneration as early events in Alzheimer’s disease: Implications to mitochondria-targeted antioxidant therapeutics. *Biochim. Biophys. Acta* 1822:639. 10.1016/J.BBADIS.2011.10.011 22037588 PMC3272314

[B61] Sanmartín C. D. Veloso P. Adasme T. Lobos P. Bruna B. Galaz J. (2017). RyR2-mediated Ca2+ release and mitochondrial ROS generation partake in the synaptic dysfunction caused by amyloid β peptide oligomers. *Front. Mol. Neurosci.* 10:115. 10.3389/FNMOL.2017.00115/BIBTEX 28487634 PMC5403897

[B62] Schaeffer E. L. Gattaz W. F. (2008). Cholinergic and glutamatergic alterations beginning at the early stages of Alzheimer disease: Participation of the phospholipase A2 enzyme. *Psychopharmacology* 198 1–27. 10.1007/s00213-008-1092-0 18392810

[B63] Schliebs R. Arendt T. (2011). The cholinergic system in aging and neuronal degeneration. *Behav. Brain Res.* 221 555–563. 10.1016/j.bbr.2010.11.058 21145918

[B64] Selkoe D. J. (2002). Alzheimer’s disease is a synaptic failure. *Science* 298 789–791. 10.1126/science.1074069 12399581

[B65] Shankar G. M. Walsh D. M. (2009). Alzheimer’s disease: Synaptic dysfunction and Aβ. *Mol. Neurodegener.* 4:48. 10.1186/1750-1326-4-48 19930651 PMC2788538

[B66] Stine W. B. Dahlgren K. N. Krafft G. A. LaDu M. J. (2003). In vitro characterization of conditions for amyloid-β peptide oligomerization and fibrillogenesis. *J. Biol. Chem.* 278 11612–11622. 10.1074/jbc.M210207200 12499373

[B67] Terni B. Boada J. Portero-Otin M. Pamplona R. Ferrer I. (2010). Mitochondrial ATP-synthase in the entorhinal cortex is a target of oxidative stress at stages I/II of alzheimer’s disease pathology. *Brain Pathol.* 20 222–233. 10.1111/j.1750-3639.2009.00266.x 19298596 PMC8094794

[B68] Timón-Gómez A. Nývltová E. Abriata L. A. Vila A. J. Hosler J. Barrientos A. (2018). Mitochondrial cytochrome c oxidase biogenesis: Recent developments. *Semin. Cell Dev. Biol.* 76:163. 10.1016/J.SEMCDB.2017.08.055 28870773 PMC5842095

[B69] Tönnies E. Trushina E. (2017). Oxidative stress, synaptic dysfunction, and Alzheimer’s disease. *J. Alzheimers Dis.* 57:1105. 10.3233/JAD-161088 28059794 PMC5409043

[B70] Tu S. Okamoto S.-I. Lipton S. A. Xu H. (2014). Oligomeric Aβ-induced synaptic dysfunction in Alzheimer’s disease. *Mol. Neurodegener.* 9:48. 10.1186/1750-1326-9-48 25394486 PMC4237769

[B71] Uttara B. Singh A. Zamboni P. Mahajan R. (2009). Oxidative stress and neurodegenerative diseases: A review of upstream and downstream antioxidant therapeutic options. *Curr. Neuropharmacol.* 7 65–74. 10.2174/157015909787602823 19721819 PMC2724665

[B72] van Hoesen G. W. Hyman B. T. Damasio A. R. (1991). Entorhinal cortex pathology in Alzheimer’s disease. *Hippocampus* 1 1–8. 10.1002/hipo.450010102 1669339

[B73] Valla J. Berndt J. D. Gonzalez-Lima F. (2001). Energy hypometabolism in posterior cingulate cortex of Alzheimer’s patients: Superficial laminar cytochrome oxidase associated with disease duration. *J. Neurosci.* 21:4923. 10.1523/JNEUROSCI.21-13-04923.2001 11425920 PMC6762373

[B74] Velayudhan L. Proitsi P. Westman E. Muehlboeck J. S. Mecocci P. Vellas B. (2013). Entorhinal cortex thickness predicts cognitive decline in Alzheimer’s disease. *J. Alzheimers Dis.* 33 755–766. 10.3233/JAD-2012-121408 23047370

[B75] Wang X. Michaelis E. K. (2010). Selective neuronal vulnerability to oxidative stress in the brain. *Front. Aging Neurosci.* 2:12. 10.3389/fnagi.2010.00012 20552050 PMC2874397

[B76] Wang W. Zhao F. Ma X. Perry G. Zhu X. (2020). Mitochondria dysfunction in the pathogenesis of Alzheimer’s disease: Recent advances. *Mol. Neurodegener.* 15:30 10.1186/S13024-020-00376-6 32471464 PMC7257174

[B77] Wang X. Wang W. Li L. Perry G. Lee H. G. Zhu X. (2014). Oxidative stress and mitochondrial dysfunction in Alzheimer’s disease. *Biochim. Biophys. Acta* 1842 1240–1247. 10.1016/j.bbadis.2013.10.015 24189435 PMC4007397

[B78] Wogulis M. Wright S. Cunningham D. Chilcote T. Powell K. Rydel R. E. (2005). Nucleation-dependent polymerization is an essential component of amyloid-mediated neuronal cell death. *J. Neurosci.* 25 1071–1080. 10.1523/JNEUROSCI.2381-04.2005 15689542 PMC6725948

[B79] Xiao T. Jiao B. Zhang W. Pan C. Wei J. Liu X. (2017). Identification of CHCHD10 mutation in chinese patients with Alzheimer disease. *Mol. Neurobiol.* 54 5243–5247. 10.1007/s12035-016-0056-3 27578015

[B80] Yu Q. Wang Y. Du F. Yan S. Hu G. Origlia N. (2018). Overexpression of endophilin A1 exacerbates synaptic alterations in a mouse model of Alzheimer’s disease. *Nat. Commun.* 9:2968. 10.1038/S41467-018-04389-0 30061577 PMC6065365

[B81] Zhou M. Zhang F. Zhao L. Qian J. Dong C. (2016). Entorhinal cortex: A good biomarker of mild cognitive impairment and mild Alzheimer’s disease. *Rev. Neurosci.* 27 185–195. 10.1515/revneuro-2015-0019 26444348

